# Supplementary material for: Modeling glioblastoma heterogeneity as a dynamic network of cell states
Source: Mol Syst Biol. 2021 Sep 16;17(9):e10105. doi: 10.15252/msb.202010105 (PMC8444284; doi:10.15252/msb.202010105)
Supplement: Supplementary file 6 — Source Data for Figure 5 [file MSB-17-e10105-s004.zip › Figure5A_sourcedata/GSEA_3071/nosigdb_stateII.GseaPreranked.1622795151171/RICHARDS_ZHONG_NATURE_2018_ASTROCYTES_UPREG.html]

Details for gene set RICHARDS\_ZHONG\_NATURE\_2018\_ASTROCYTES\_UPREG[GSEA]

|  || Dataset | state23071 |
| Phenotype | NoPhenotypeAvailable |
| Upregulated in class | na\_neg |
| GeneSet | RICHARDS\_ZHONG\_NATURE\_2018\_ASTROCYTES\_UPREG |
| Enrichment Score (ES) | -0.52798045 |
| Normalized Enrichment Score (NES) | -3.112552 |
| Nominal p-value | 0.0 |
| FDR q-value | 0.0 |
| FWER p-Value | 0.0 |
Table: GSEA Results Summary

  

Fig 1: Enrichment plot: RICHARDS\_ZHONG\_NATURE\_2018\_ASTROCYTES\_UPREG      
 Profile of the Running ES Score & Positions of GeneSet Members on the Rank Ordered List

  

| PROBE | GENE SYMBOL | GENE\_TITLE | RANK IN GENE LIST | RANK METRIC SCORE | RUNNING ES | CORE ENRICHMENT || 1 | F3 |  |  | 13 | 1.555 | 0.0110 | No |
| 2 | MT1E |  |  | 106 | 1.033 | -0.0028 | No |
| 3 | MGLL |  |  | 227 | 0.848 | -0.0254 | No |
| 4 | ALCAM |  |  | 288 | 0.771 | -0.0335 | No |
| 5 | MT2A |  |  | 393 | 0.682 | -0.0536 | No |
| 6 | IGFBP4 |  |  | 401 | 0.678 | -0.0491 | No |
| 7 | PHGDH |  |  | 429 | 0.664 | -0.0498 | No |
| 8 | ZFP36L2 |  |  | 458 | 0.647 | -0.0510 | No |
| 9 | NT5E |  |  | 481 | 0.637 | -0.0507 | No |
| 10 | SLC9A3R1 |  |  | 497 | 0.630 | -0.0487 | No |
| 11 | PDLIM5 |  |  | 526 | 0.613 | -0.0502 | No |
| 12 | ALPL |  |  | 571 | 0.590 | -0.0559 | No |
| 13 | RGCC |  |  | 600 | 0.578 | -0.0577 | No |
| 14 | SNX5 |  |  | 762 | 0.512 | -0.0937 | No |
| 15 | HOPX |  |  | 768 | 0.510 | -0.0903 | No |
| 16 | ID1 |  |  | 769 | 0.509 | -0.0857 | No |
| 17 | PLEC |  |  | 796 | 0.500 | -0.0877 | No |
| 18 | RAB34 |  |  | 803 | 0.496 | -0.0846 | No |
| 19 | ADORA2B |  |  | 841 | 0.486 | -0.0895 | No |
| 20 | PLAGL1 |  |  | 892 | 0.473 | -0.0978 | No |
| 21 | RGS20 |  |  | 942 | 0.461 | -0.1060 | No |
| 22 | CYP51A1 |  |  | 952 | 0.458 | -0.1041 | No |
| 23 | SOAT1 |  |  | 982 | 0.449 | -0.1073 | No |
| 24 | DDAH1 |  |  | 1028 | 0.434 | -0.1147 | No |
| 25 | BCAP29 |  |  | 1053 | 0.427 | -0.1169 | No |
| 26 | EFHD2 |  |  | 1090 | 0.419 | -0.1221 | No |
| 27 | NOTCH3 |  |  | 1141 | 0.409 | -0.1310 | No |
| 28 | CDC42EP1 |  |  | 1193 | 0.398 | -0.1403 | No |
| 29 | SLC3A2 |  |  | 1203 | 0.396 | -0.1389 | No |
| 30 | HES4 |  |  | 1250 | 0.387 | -0.1470 | No |
| 31 | ABHD3 |  |  | 1288 | 0.380 | -0.1529 | No |
| 32 | GLUD1 |  |  | 1397 | 0.358 | -0.1769 | No |
| 33 | PSAT1 |  |  | 1505 | 0.340 | -0.2009 | No |
| 34 | PHACTR2 |  |  | 1596 | 0.324 | -0.2207 | No |
| 35 | JUN |  |  | 1725 | 0.307 | -0.2502 | No |
| 36 | SDC3 |  |  | 1908 | 0.284 | -0.2937 | No |
| 37 | FERMT2 |  |  | 2003 | 0.272 | -0.3149 | No |
| 38 | RCN1 |  |  | 2051 | 0.267 | -0.3244 | No |
| 39 | ANXA5 |  |  | 2187 | 0.253 | -0.3562 | No |
| 40 | PEA15 |  |  | 2209 | 0.251 | -0.3592 | No |
| 41 | ADGRG1 |  |  | 2239 | -0.252 | -0.3642 | No |
| 42 | TEX264 |  |  | 2267 | -0.255 | -0.3687 | No |
| 43 | LRRTM3 |  |  | 2282 | -0.257 | -0.3699 | No |
| 44 | LRP4 |  |  | 2300 | -0.259 | -0.3718 | No |
| 45 | FADS2 |  |  | 2338 | -0.263 | -0.3788 | No |
| 46 | GJA1 |  |  | 2366 | -0.266 | -0.3832 | No |
| 47 | SEMA6D |  |  | 2407 | -0.272 | -0.3908 | No |
| 48 | DTNA |  |  | 2414 | -0.272 | -0.3898 | No |
| 49 | SPRY2 |  |  | 2455 | -0.278 | -0.3974 | No |
| 50 | CRYL1 |  |  | 2460 | -0.279 | -0.3958 | No |
| 51 | HADHB |  |  | 2489 | -0.283 | -0.4003 | No |
| 52 | SHISA4 |  |  | 2493 | -0.284 | -0.3985 | No |
| 53 | GATM |  |  | 2501 | -0.285 | -0.3976 | No |
| 54 | EMC2 |  |  | 2512 | -0.287 | -0.3975 | No |
| 55 | DIO2 |  |  | 2599 | -0.301 | -0.4165 | No |
| 56 | TMEM132B |  |  | 2623 | -0.304 | -0.4196 | No |
| 57 | EFEMP2 |  |  | 2654 | -0.309 | -0.4243 | No |
| 58 | PNPLA3 |  |  | 2699 | -0.316 | -0.4325 | No |
| 59 | MFGE8 |  |  | 2712 | -0.319 | -0.4327 | No |
| 60 | FAM84B |  |  | 2713 | -0.319 | -0.4297 | No |
| 61 | ZFYVE21 |  |  | 2758 | -0.328 | -0.4378 | No |
| 62 | LEPROT |  |  | 2761 | -0.328 | -0.4353 | No |
| 63 | METTL7A |  |  | 2818 | -0.339 | -0.4464 | No |
| 64 | SIRPA |  |  | 2823 | -0.340 | -0.4443 | No |
| 65 | TSC22D4 |  |  | 2847 | -0.344 | -0.4470 | No |
| 66 | C3orf70 |  |  | 2851 | -0.345 | -0.4446 | No |
| 67 | S100A13 |  |  | 2858 | -0.346 | -0.4429 | No |
| 68 | SLITRK2 |  |  | 2970 | -0.368 | -0.4676 | No |
| 69 | SNX3 |  |  | 2987 | -0.370 | -0.4683 | No |
| 70 | APBB2 |  |  | 3023 | -0.378 | -0.4736 | No |
| 71 | ALDH6A1 |  |  | 3027 | -0.379 | -0.4709 | No |
| 72 | PHYHIPL |  |  | 3049 | -0.385 | -0.4727 | No |
| 73 | ABAT |  |  | 3075 | -0.391 | -0.4754 | No |
| 74 | BMP7 |  |  | 3127 | -0.407 | -0.4846 | No |
| 75 | HEY1 |  |  | 3130 | -0.408 | -0.4814 | No |
| 76 | PLEKHB1 |  |  | 3131 | -0.409 | -0.4776 | No |
| 77 | BDH2 |  |  | 3162 | -0.418 | -0.4814 | No |
| 78 | PON2 |  |  | 3216 | -0.432 | -0.4908 | No |
| 79 | SOX9 |  |  | 3230 | -0.435 | -0.4901 | No |
| 80 | TSPAN6 |  |  | 3240 | -0.438 | -0.4884 | No |
| 81 | S100A16 |  |  | 3338 | -0.472 | -0.5086 | No |
| 82 | MRC2 |  |  | 3391 | -0.494 | -0.5172 | No |
| 83 | DOK5 |  |  | 3409 | -0.503 | -0.5169 | No |
| 84 | TRIL |  |  | 3411 | -0.503 | -0.5125 | No |
| 85 | SEMA6A |  |  | 3455 | -0.521 | -0.5186 | No |
| 86 | QKI |  |  | 3467 | -0.526 | -0.5166 | No |
| 87 | FABP5 |  |  | 3513 | -0.549 | -0.5229 | Yes |
| 88 | ITM2C |  |  | 3518 | -0.552 | -0.5189 | Yes |
| 89 | AASS |  |  | 3539 | -0.564 | -0.5188 | Yes |
| 90 | CD9 |  |  | 3567 | -0.581 | -0.5203 | Yes |
| 91 | IFITM3 |  |  | 3578 | -0.589 | -0.5174 | Yes |
| 92 | DBI |  |  | 3597 | -0.601 | -0.5164 | Yes |
| 93 | SPATA6 |  |  | 3606 | -0.608 | -0.5129 | Yes |
| 94 | AIF1L |  |  | 3621 | -0.617 | -0.5108 | Yes |
| 95 | PBXIP1 |  |  | 3635 | -0.633 | -0.5082 | Yes |
| 96 | C1orf122 |  |  | 3638 | -0.634 | -0.5029 | Yes |
| 97 | MEIS1 |  |  | 3651 | -0.648 | -0.5000 | Yes |
| 98 | MLC1 |  |  | 3658 | -0.652 | -0.4956 | Yes |
| 99 | DAG1 |  |  | 3662 | -0.654 | -0.4903 | Yes |
| 100 | ID2 |  |  | 3664 | -0.654 | -0.4846 | Yes |
| 101 | LITAF |  |  | 3670 | -0.660 | -0.4798 | Yes |
| 102 | OLFM2 |  |  | 3690 | -0.672 | -0.4784 | Yes |
| 103 | STON2 |  |  | 3702 | -0.689 | -0.4749 | Yes |
| 104 | PREX1 |  |  | 3716 | -0.698 | -0.4718 | Yes |
| 105 | FAT1 |  |  | 3730 | -0.708 | -0.4686 | Yes |
| 106 | HTRA1 |  |  | 3741 | -0.719 | -0.4645 | Yes |
| 107 | GNG7 |  |  | 3746 | -0.724 | -0.4589 | Yes |
| 108 | RAB31 |  |  | 3757 | -0.737 | -0.4547 | Yes |
| 109 | NCAN |  |  | 3763 | -0.748 | -0.4491 | Yes |
| 110 | PRCP |  |  | 3775 | -0.766 | -0.4448 | Yes |
| 111 | TTYH1 |  |  | 3807 | -0.795 | -0.4454 | Yes |
| 112 | SOX8 |  |  | 3809 | -0.797 | -0.4383 | Yes |
| 113 | WSCD1 |  |  | 3817 | -0.803 | -0.4327 | Yes |
| 114 | APOE |  |  | 3864 | -0.860 | -0.4365 | Yes |
| 115 | DDR1 |  |  | 3903 | -0.948 | -0.4374 | Yes |
| 116 | TM7SF2 |  |  | 3918 | -0.999 | -0.4318 | Yes |
| 117 | BCHE |  |  | 3926 | -1.012 | -0.4242 | Yes |
| 118 | ETV1 |  |  | 3934 | -1.026 | -0.4166 | Yes |
| 119 | GPRC5B |  |  | 3937 | -1.032 | -0.4076 | Yes |
| 120 | SLC1A3 |  |  | 3942 | -1.040 | -0.3991 | Yes |
| 121 | TRIB2 |  |  | 3944 | -1.041 | -0.3898 | Yes |
| 122 | ITPR2 |  |  | 3946 | -1.044 | -0.3805 | Yes |
| 123 | GNG11 |  |  | 3952 | -1.062 | -0.3720 | Yes |
| 124 | SOX2 |  |  | 3953 | -1.069 | -0.3622 | Yes |
| 125 | ETV5 |  |  | 3971 | -1.116 | -0.3563 | Yes |
| 126 | CD82 |  |  | 3973 | -1.119 | -0.3463 | Yes |
| 127 | NPNT |  |  | 3989 | -1.189 | -0.3392 | Yes |
| 128 | MYO10 |  |  | 3990 | -1.193 | -0.3282 | Yes |
| 129 | KLHDC8A |  |  | 4005 | -1.301 | -0.3198 | Yes |
| 130 | FAM181B |  |  | 4037 | -1.542 | -0.3135 | Yes |
| 131 | TNC |  |  | 4039 | -1.549 | -0.2996 | Yes |
| 132 | SLC1A2 |  |  | 4040 | -1.555 | -0.2853 | Yes |
| 133 | SPRY1 |  |  | 4044 | -1.573 | -0.2716 | Yes |
| 134 | PDPN |  |  | 4046 | -1.612 | -0.2571 | Yes |
| 135 | LRRN3 |  |  | 4064 | -1.810 | -0.2448 | Yes |
| 136 | CXCL14 |  |  | 4066 | -1.885 | -0.2278 | Yes |
| 137 | CLU |  |  | 4069 | -1.908 | -0.2108 | Yes |
| 138 | SCRG1 |  |  | 4070 | -2.021 | -0.1922 | Yes |
| 139 | PTN |  |  | 4077 | -2.187 | -0.1737 | Yes |
| 140 | ID4 |  |  | 4079 | -2.308 | -0.1528 | Yes |
| 141 | GFAP |  |  | 4080 | -2.343 | -0.1313 | Yes |
| 142 | S100B |  |  | 4081 | -2.383 | -0.1094 | Yes |
| 143 | GPM6B |  |  | 4083 | -2.435 | -0.0873 | Yes |
| 144 | C1orf61 |  |  | 4084 | -2.474 | -0.0646 | Yes |
| 145 | FABP7 |  |  | 4095 | -3.351 | -0.0364 | Yes |
| 146 | PTPRZ1 |  |  | 4097 | -4.027 | 0.0003 | Yes |
Table: GSEA details [plain text format]

  

Fig 2: RICHARDS\_ZHONG\_NATURE\_2018\_ASTROCYTES\_UPREG: Random ES distribution      
 Gene set null distribution of ES for **RICHARDS\_ZHONG\_NATURE\_2018\_ASTROCYTES\_UPREG**
